# Estrogen α and β Receptor Expression in the Various Regions of Resected Glioblastoma Multiforme Tumors and in an In Vitro Model

**DOI:** 10.3390/ijms25074130

**Published:** 2024-04-08

**Authors:** Donata Simińska, Klaudyna Kojder, Dariusz Jeżewski, Maciej Tarnowski, Patrycja Tomasiak, Katarzyna Piotrowska, Agnieszka Kolasa, Kapczuk Patrycja, Dariusz Chlubek, Irena Baranowska-Bosiacka

**Affiliations:** 1 Department of Biochemistry and Medical Chemistry, Pomeranian Medical University in Szczecin, Powstańców Wlkp. 72, 70-111 Szczecin, Poland; donata.siminska@pum.edu.pl (D.S.); patrycja.kapczuk@pum.edu.pl (K.P.); irena.baranowska.bosiacka@pum.edu.pl (I.B.-B.); 2 Department of Anaesthesiology and Intensive Care, Pomeranian Medical University in Szczecin, Unii Lubelskiej 1, 71-252 Szczecin, Poland; klaudyna.kojder@pum.edu.pl; 3 Department of Neurosurgery and Pediatric Neurosurgery, Pomeranian Medical University in Szczecin, Unii Lubelskiej 1, 71-252 Szczecin, Poland; dariusz.jezewski@pum.edu.pl; 4 Department of Applied Neurocognitivistics, Pomeranian Medical University in Szczecin, Unii Lubelskiej 1, 71-252 Szczecin, Poland; 5 Department of Physiology in Health Sciences, Pomeranian Medical University in Szczecin, Żołnierska 54, 70-210 Szczecin, Poland; maciej.tarnowski@pum.edu.pl; 6 Institute of Physical Culture Sciences, University of Szczecin, 70-453 Szczecin, Poland; patrycja.tomasiak@usz.edu.pl; 7 Department of Physiology, Pomeranian Medical University in Szczecin, Powstańców Wlkp. 72, 70-111 Szczecin, Poland; katarzyna.piotrowska@pum.edu.pl; 8 Department of Histology and Embryology, Pomeranian Medical University in Szczecin, Powstańców Wlkp. 72, 70-111 Szczecin, Poland; agnieszka.kolasa@pum.edu.pl

**Keywords:** estrogen receptor α, estrogen receptor β, glioblastoma multiforme, hypoxia, nutrient deficiency, nuclear sex hormone receptors

## Abstract

Glioblastoma multiforme (GBM) is a malignant tumor with a higher prevalence in men and a higher survival rate in transmenopausal women. It exhibits distinct areas influenced by changing environmental conditions. This study examines how these areas differ in the levels of estrogen receptors (ERs) which play an important role in the development and progression of many cancers, and whose expression levels are often correlated with patient survival. This study utilized two research models: an in vitro model employing the U87 cell line and a second model involving tumors resected from patients (including tumor core, enhancing tumor region, and peritumoral area). ER expression was assessed at both gene and protein levels, with the results validated using confocal microscopy and immunohistochemistry. Under hypoxic conditions, the U87 line displayed a decrease in ERβ mRNA expression and an increase in ERα mRNA expression. In patient samples, ERβ mRNA expression was lower in the tumor core compared to the enhancing tumor region (only in males when the study group was divided by sex). In addition, ERβ protein expression was lower in the tumor core than in the peritumoral area (only in women when the study group was divided by sex). Immunohistochemical analysis indicated the highest ERβ protein expression in the enhancing tumor area, followed by the peritumoral area, and the lowest in the tumor core. The findings suggest that ER expression may significantly influence the development of GBM, exhibiting variability under the influence of conditions present in different tumor areas.

## 1. Introduction

Glioblastoma multiforme (GBM) is one of the most common malignant tumors of the central nervous system [1]. Most commonly diagnosed in the elderly and in men, it has the highest incidence rate (3.27 per 100,000 population) among malignant brain tumors [1,2,3]. The median survival rate for patients with GBM is only 15 months [4,5] and a 5-year survival occurs in 5.5% of patients [2]. 

Early diagnosis of GBM tumors is difficult due to the non-specificity of symptoms and the older average age of patients. Treatment of patients with GBM is also challenging due to the high degree of proliferation, infiltrative nature, and significant cellular heterogeneity of the tumor [6]. In the case of GBM and other diffuse gliomas, complete surgical resection is hindered by their extensive infiltration into the CNS parenchyma [7]. In addition, the high inter- and intra-tumor heterogeneity makes it very difficult to correctly identify therapeutic targets [6].

GBM tumors include not only cancer cell lines that differ in expression and isoform selection [8], but also a wide range of non-cancerous stromal cells, including blood vessels, various infiltrating and resident immune cells, and other glial cell types [9]. Also included in the tumor cell pool are cancer stem cells (CSCs), which result in even greater tumor heterogeneity and proliferative potential [10]. In addition to cellular variability, another characteristic feature of GBM is the variability in the tumor microenvironment induced by differences in the access to nutrients and oxygen caused by the massive growth and abnormal angiogenesis of the tumor [9]. This affects the molecular profile of tumor cells and influences the formation of different GBM tumor areas, i.e., the hypoxic non-growing core of the tumor (tumor core), the region of intense tumor growth occurring around the vasculature (enhancing tumor region), and the tumor periphery (peritumoral area) that heavily infiltrates the CNS near the tumor [9].

The higher incidence in men [3,11,12] and the higher survival rate in premenopausal women [13,14,15,16] may be due to the different molecular profiles of GBM tumors in these groups and may indicate the influence of nuclear sex hormone receptors, namely estrogen receptors.

Estrogen receptor α (ERα/NR3A1 (nuclear receptor subfamily 3, group A, member 1) [17] was the first discovered estrogen receptor [18]. ERα is encoded by the *ESR1* gene located at position 6q25.1-q25.2 [19], containing 140 Kb [20], of which 1785 bp [21] are 8 exons present in the gene [20]. ERα consists of 595 amino acids and has a total molecular weight of 66.2 kDa [21]. Estrogen receptor beta (ERβ), also known as nuclear receptor subfamily 3, group A, member 2 (NR3A2), was discovered 38 years after ERα. The gene encoding ERβ (*ESR2*) is located at position 14q22-q24 [18]. Like ERα, ERβ comprises 8 exons with a total length of 40 Kb [18]. It consists of 530 amino acids and has a molecular weight of 59.2 kDa [22].

Estrogen receptors α and β differ in their expression profiles in different tissues of the human body. Often, one of them is expressed at higher levels than the other. ERα is mainly expressed in female reproductive tissues (uterus, ovary), breast, kidney, bone, white adipose tissue, and liver, whereas ERβ expression is found in the ovary, central nervous system, cardiovascular system, lung, male reproductive organs, prostate, colon, kidney, and immune system [23]. The receptors regulate many physiological processes, such as the development and proper functioning of the female reproductive system or the maintenance of skeletal homeostasis and regulation of metabolism [24].

ERα promotes cell growth and proliferation, thus contributing to tumor growth [24]. Also well known is the involvement of ERα signaling in breast cancer progression [25]. ERα is expressed in 70% of breast tumors [26], and many drugs such as tamoxifen [27] and fulvestrant [28] target this receptor. 

ERα is also involved in the development of cancers such as prostate cancer, oral squamous cell carcinoma, and endometrial cancer. In prostate cancer, tumor growth is stimulated by estrogen acting through ERα, the blocking of which is associated with reduced bone or lung metastasis [29]. In oral squamous cell carcinoma, ERα expression is found more frequently in older male patients, and the presence of this receptor is often associated with the malignancy of this cancer [30]. ERα expression is also detected in endometrial cancer cells and is reported to be elevated in endometrial cancers that result in lymph node metastasis [31]. In liver cancer, both estrogen and ERα appear to play a protective and tumor progression-limiting role [32].

ERβ is known as a tumor suppressor. In ovarian cancer, ERβ expression levels are lower than in healthy tissue [33], and the loss of expression of this receptor is associated with shorter overall survival of cancer patients [34]. This effect is not only related to the amount of the receptor but also its localization in the cell. For example, in ovarian cancer cells, ERβ is observed mainly in the cytoplasm, while in normal cells it is found in the nucleus [35]. 

In breast cancer, a decrease in ERβ expression is observed as the disease progresses [36]. The re-expression of this receptor is associated with a decrease in cell proliferation as a result of suppression of the MAPK (mitogen-activated protein kinase) and PI3K (phosphoinositide 3-kinases) signaling pathways [37], promotion of apoptosis, and improved response to chemotherapy [38]. In prostate cancer, ERβ also shows increasingly low expression with tumor progression as a result of the methylation of its promoter [39]. A protective role for ERβ has been demonstrated in colon cancer, as ERβ contributes to the inhibition of inflammatory processes involved in colon cancer carcinogenesis [40].

To date, studies of estrogen receptors in glioma have focused on tumors from patients or laboratory animals and cell lines. However, studies on tumors have usually treated the cancer tumor as a whole, while cell cultures do not capture the diversity and spatial variability of cell lines in a tumor. 

The aim of our work was to determine the differences in estrogen receptor expression between the three areas of the tumor, namely the tumor core, enhancing tumor region, and peritumoral area.

## 2. Results

### 2.1. Changes in ERα Gene and Protein Expression in U87 Line Cells Cultured under Different Test Conditions

**Figure 1:**
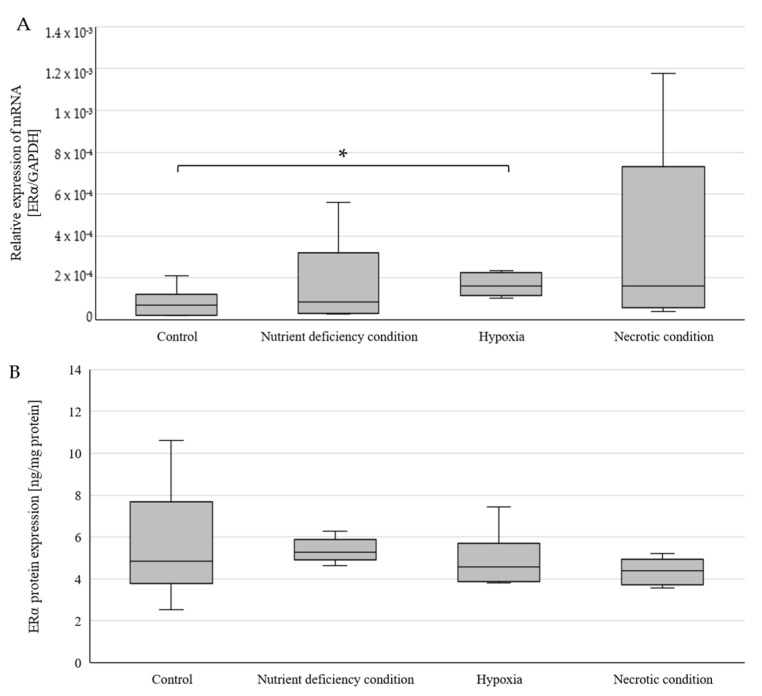
Expression of *ERα* gene (**A**) and ERα protein (**B**) in U87 cells cultured under different conditions. Data are representative of each group cultured in control, nutrient-deficient, hypoxic, and necrotic conditions. Statistical analysis was performed using *t*-test *, *p* < 0.005.

**Figure 2:**
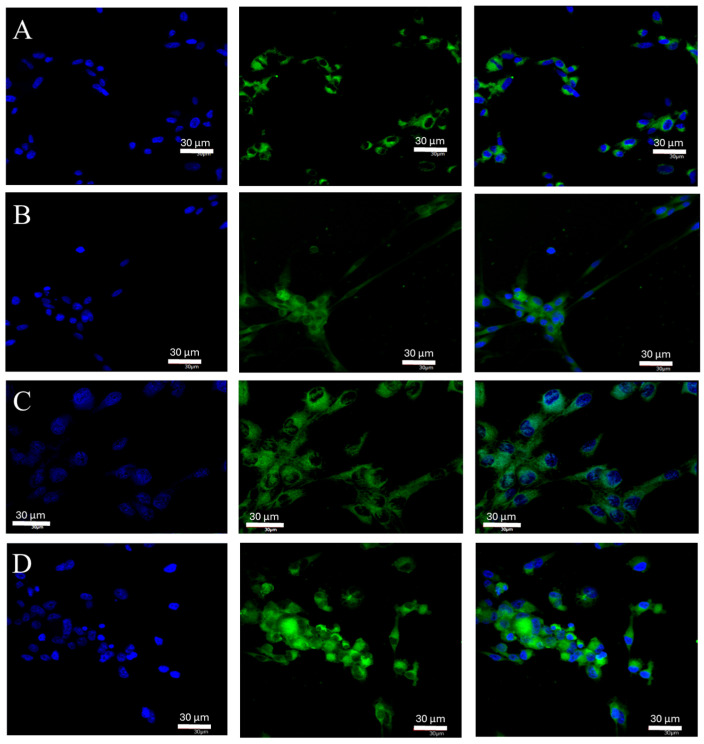
Representative images taken with the FV1000 confocal microscope system (Olympus, Hamburg, Germany) show *ERα* protein expression in U87 cells cultured under specific conditions: control (**A**), nutrient deficiency (**B**), hypoxia (**C**), and necrotic conditions (**D**). FITC (AR) and DAPI (nuclear) markers were used. Microphotographs were taken at ×20 magnification (**A**,**B**,**D**) and ×40 magnification (**C**); scale bar 30 µm.

An average 300% statistically significant increase (*p* = 0.002827) in *ERα* mRNA expression was observed in cells cultured under hypoxia compared to cells cultured under control conditions (Figure 1A). No significant differences were observed for nutrient deficiency and necrotic conditions. ERα protein was expressed at similar levels in all culture conditions tested and no statistically significant changes were observed (Figure 1B). The average expression of ERα protein for all conditions tested was 5.07 ng/mg of total protein. Analysis of cell culture images captured by a confocal microscopy system led to similar conclusions. In the case of ERα, luminescence was also observed throughout the cell volume with enhancement near the nucleus (Figure 2).

### 2.2. Changes in ERα Gene and Protein Expression in Individual GBM Tumoral Areas Obtained from Patients

**Figure 3:**
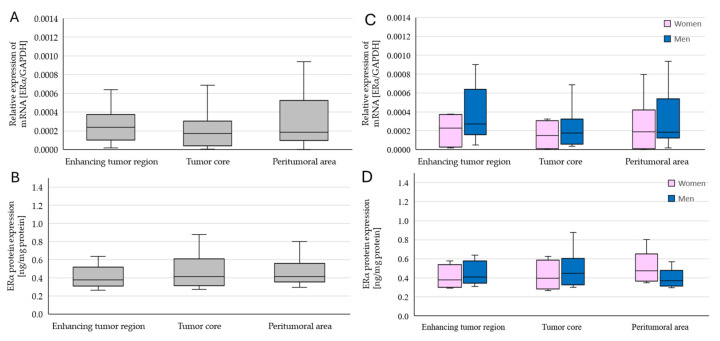
*ERα* gene (**A**,**C**) and ERα protein (**B**,**D**) expression in individual GBM tumoral areas obtained from patients. Data are representative of individual tumoral areas (tumor core, enhancing tumor region, and peritumoral area) in the entire group of patients (**A**,**B**) and by sex (**C**,**D**). Statistical analysis was performed using the Wilcoxon signed-rank test.

**Figure 4:**
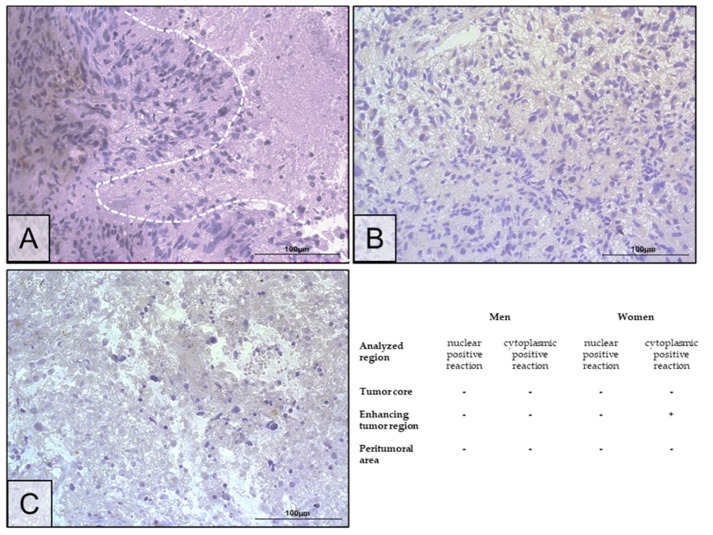
Representative microphotographs show ERα protein expression in the tumor core (the necrotic area is circled with a white dotted line) (**A**), enhancing tumor region (**B**), and peritumoral area (**C**) of a tumor diagnosed as GBM. Microphotographs were taken at ×40 magnification; scale bar 100 µm. The + sign indicates the presence of a positive immunohistochemical reaction, and the - sign indicates its absence.

The expression of *ERα* mRNA (Figure 3A,C) and ERα protein (Figure 3B,D) did not differ between tumoral areas among men and women, as well as between sexes within a single tumoral area. The average expression level of *ERα* mRNA was 0.00025 [*ERα/GAPDH*], while that of ERα protein was 0.45 ng/mg of total protein. In immunohistochemistry, there was no expression of ERα receptor protein in the tumor core, enhancing tumor region, and peritumoral area (Figure 4).

### 2.3. Changes in ERβ Gene and Protein Expression in U87 Line Cells Cultured under Different Test Conditions

**Figure 5:**
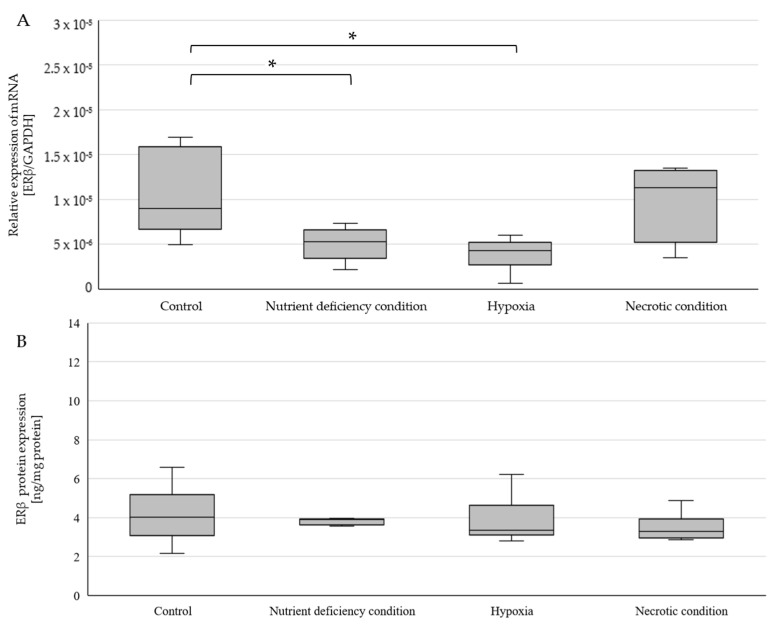
Expression of the *ERβ* gene (**A**) and ERβ protein (**B**) in U87 cells cultured under different conditions. Data are representative of each group cultured in control, nutrient-deficient, hypoxic, and necrotic conditions. Statistical analysis was performed using a *t*-test * *p* < 0.05.

**Figure 6:**
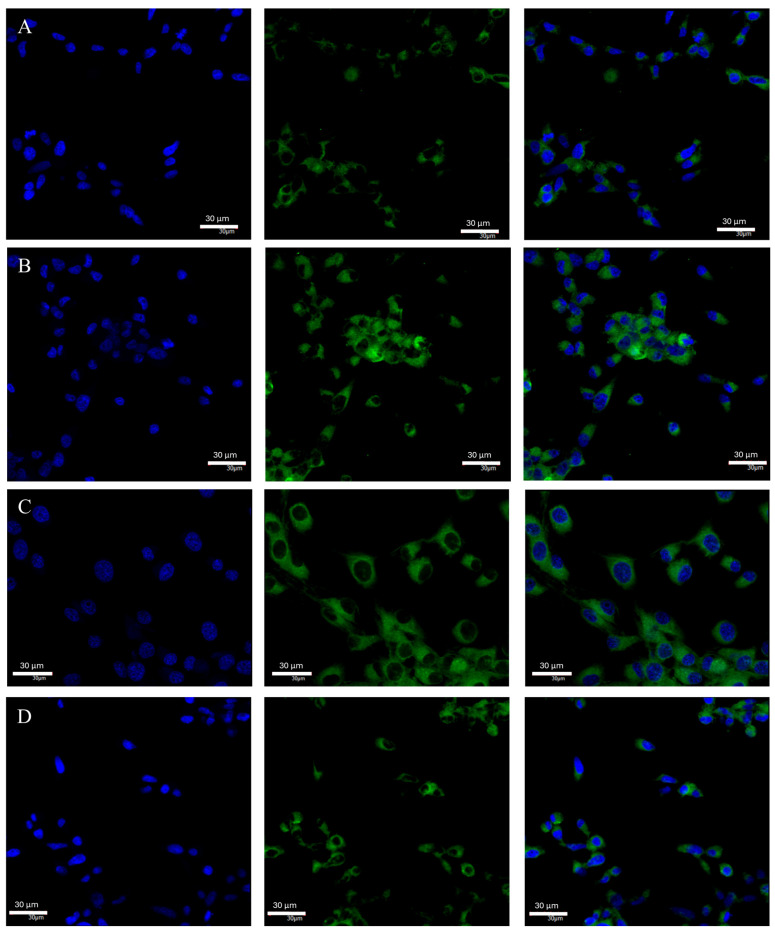
Representative images taken with the FV1000 confocal microscope system (Olympus, Hamburg, Germany) show ERβ protein expression in U87 cells cultured under specific conditions: control (**A**), nutrient deficiency (**B**), hypoxia (**C**), and necrotic conditions (**D**). FITC (AR) and DAPI (nuclear) markers were used. Microphotographs were taken at ×20 magnification (**A**,**B**,**D**) and ×40 magnification (**C**); scale bar 30 µm.

There was a statistically significant decrease in *ERβ* mRNA expression under both hypoxia (*p* = 0.026203) and nutrient-deficient conditions (*p* = 0.028163) (Figure 5A). In both cases, *ERβ* mRNA expression averaged about 50% of that of controls. No changes in the mRNA expression of this receptor were observed under necrotic conditions. There were also no changes in ERβ protein expression between the different test conditions (Figure 5B). The average expression of ERβ protein for all test conditions was 3.82 ng/mg of total protein. Confocal microscopy confirmed the results obtained by ELISA. The degree of luminescence did not differ between the different test conditions (Figure 6). The highest intensity of luminescence was localized around the cell nucleus but also occurred in the cytoplasm of the cells.

### 2.4. Changes in ERβ Gene and Protein Expression in Individual GBM Tumoral Areas Obtained from Patients

**Figure 7:**
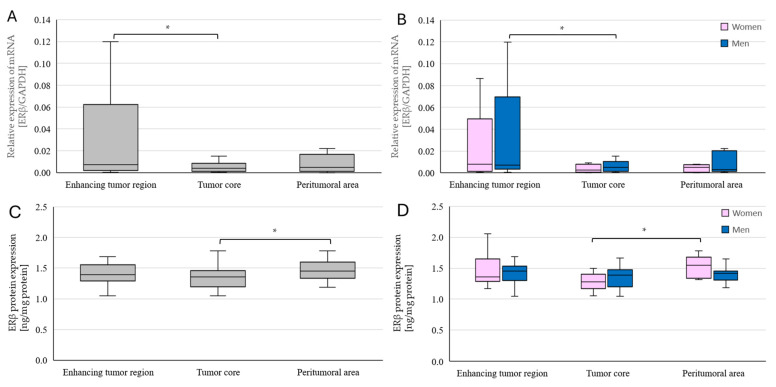
*ERβ* gene (**A**,**C**) and ERβ protein (**B**,**D**) expression in individual GBM tumoral areas obtained from patients. Data are representative of individual tumoral areas (tumor core, enhancing tumor region, and peritumoral area) in the entire group of patients (**A**,**B**) and by sex (**C**,**D**). Statistical analysis was performed using the Wilcoxon signed-rank test, * *p* < 0.05.

A statistically significant difference was also observed for the expression of the gene encoding *ERβ*. Before accounting for sex, *ERβ* mRNA expression in the enhancing tumor region was approximately 564% higher than in the tumor core (*p* = 0.035465) (Figure 7A). When sex was included in the analysis, *ERβ* mRNA expression in men was higher in the enhancing tumor area than in the tumor core, by 429% on average (*p* = 0.042523) (Figure 7B). ERβ protein expression was statistically significantly different between the tumor core and peritumoral area when not adjusted by sex, with a higher expression of about 109% noted in the peritumoral area (*p* = 0.013871) (Figure 7C). When sex was taken into account, this difference was observed only in women, with expression approximately 115% higher (*p* = 0.017291) (Figure 7D).

**Figure 8:**
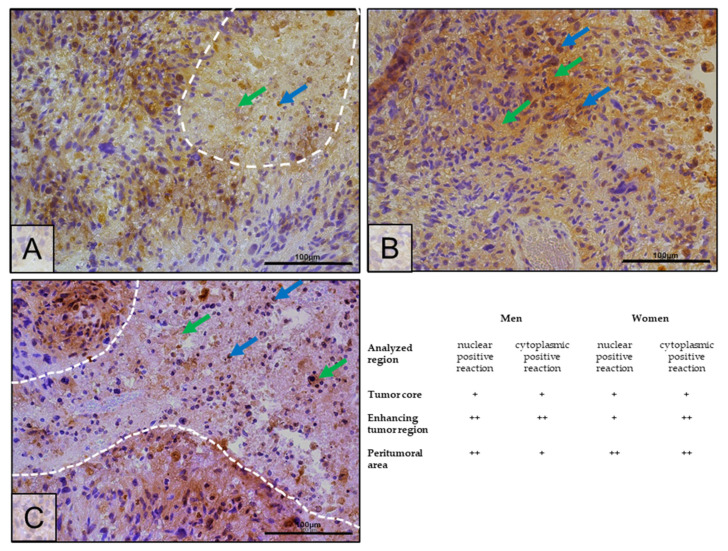
Representative microphotographs show ERβ protein expression in the tumor core (the necrotic area is circled with a white dotted line) (**A**), enhancing tumor region (**B**), and peritumoral area (**C**) of a tumor diagnosed as GBM. A positive IHC reaction result is marked with arrows: blue—nuclear positive reaction, green—cytoplasmic positive reaction. Microphotographs were taken at ×40 magnification; scale bar 100 µm. The occurrence of a positive immunohistochemical reaction is marked with the + sign, and its higher degree with the ++ sign.

Immunohistochemical analysis noted higher expression of ERβ receptor protein in the enhancing tumor region than in the tumor core (Figure 8). Expression in the enhancing tumor area was seen mainly in the cytoplasm of the cells, although it was also present in the cell nucleus (Figure 8B, green and blue arrows). In the peritumoral area, expression was at a lower level than in the enhancing tumor region, which was not shown by the results from other tests (Figure 8C). Similar to the ELISA results, ERβ receptor protein expression was lower in the tumor core than in the peritumoral area (Figure 8A,C).

## 3. Discussion

### 3.1. Estrogen Receptor α in an In Vitro Model

In our study, we noted that *ERα* mRNA expression was higher under hypoxia conditions in the U87 cell line, while there were no differences in expression at the protein level. There was also no difference in the degree of luminescence and cellular localization of ERα protein between the conditions tested.

There are many conflicting reports in the scientific literature regarding ERα expression in glioma. Initially, *ERα* mRNA expression was demonstrated in C6 (rat GBM line) and U373MG and T98G (human GBM lines) [41]. Subsequent reports contradicted these findings by showing that U87, T98G, U251MG, U373MG, U138MG, and WS1088 cell lines do not express *ERα* or *ERβ* mRNA [42]. The next study of glioma cell lines showed a lack of ERα protein expression in T98G, U87, LN229, U138, M059J, and M059K [43].

Recent studies have confirmed ERα expression in U87 and U251-MG cell lines [44,45,46]. However, it has been reported that *ERα* mRNA expression in human GBM cell lines is lower than in normal human astrocytes (GBM lines: U251, U87, T98G, and LN229) [47] and noticeably higher in U251 [47]. At the protein level, ERα is characterized by higher expression than ERβ in all the GBM lines tested in that study [47].

The discrepancies between studies on ERα expression can be explained by technological developments and increased accuracy of methods. In the work presented here, the mRNA and protein expression of ERα was confirmed in the U87 cell line. The presence of the protein was detected by two assay methods, allowing not only the quantification of the expression status of the ERα protein, but also the visualization of its distribution in the cell. *ERα* mRNA and protein expression was higher than for *ERβ*.

There are also conflicting reports in the literature about the role of ERα agonists on its expression and cell proliferation and viability. ER agonists have been shown to increase cell proliferation, as well as ERα and ERβ protein expression [44]. Another study presented a reduction in ERα expression in U87 and U251-MG cell lines following administration of 17β-estradiol [46]. It has also been reported that treatment with high concentrations of estradiol results in the lower viability of GBM cell lines LN229 and LN18 [45]; the cells presented significantly greater sensitivity to temozolomide (TMZ) after pretreatment with estradiol [45]. An ERα agonist (propylpyrazole triol) was shown to induce growth in stellate cell lines (U373 and D54) [48].

In a study on the U87 and U251 cell lines, both expressed ERα-36, with stronger expression noted in the U87 line [49]. In both these cell lines, the expression of ERα was lower than that of its isoform ERα-36. In another study, ERα-36 was expressed in both lines at both the gene and protein levels, with negligible protein expression for ERα, and mRNA expression found only in the U251 line [50].

In light of the studies described above, it is difficult to unequivocally attribute the role of ERα as a pro-oncogenic receptor in GBM. However, the prooncogenic properties of ERα [51]. Reports of ERα’s involvement in the activation of the epithelial–mesenchymal transition involved in GBM malignancy also seem relevant here [47], as 17β-estradiol and a selective ERα agonist induced an increase in the expression of mesenchymal markers such as vimentin and N-cadherin, and increased migration and invasion of GBM cells [47].

In the presented study, it was shown that hypoxia can increase ERα expression and thus presumably increase cell proliferation in the hypoxic region of the tumor. These results are consistent with the literature data [52]. However, ERα may contribute to the activation of the HIF-1 degradation pathway [53], thereby abolishing the effect of hypoxia on the cell and blocking the activation of defense mechanisms associated with hypoxic status. It should also be noted that the observed increase in *ERα* expression occurred only for mRNA expression. This could be explained by a possible autocontrol of expression through compensatory mechanisms, or by too short a period of incubation with the hypoxia-inducing agent. This hypothesis could be confirmed by repetition at other time points.

### 3.2. Estrogen Receptor α in Tumors Taken from Patients

In the work presented here, *ERα* mRNA and protein expression did not differ between glioma tumoral areas, either in the entire study group or when sex was taken into account. Immunohistochemistry showed no protein expression of ERα in the tumor core and peritumoral area, and it was locally present in the enhancing tumor region.

Lower levels of ERα expression were observed in GBM and low-grade gliomas compared to healthy tissue [47]. In addition, ERα expression was higher in GBM than in low-grade gliomas [47].

According to Dueñas Jiménez et al., stellate tumors had similar levels of ERα expression in men and women. They also showed a negative correlation of ERα expression with the degree of tumor malignancy and a positive correlation with patient survival. Expression was lower in GBM tumors than in astrocytoma [54].

GBM tumors have been shown to differ in the magnitude of ERα expression [45]. In a study by Hernández-Vega et al., higher levels of ERα and ERβ expression in GBM were associated with a poor prognosis for the patient, which is puzzling in light of the lower expression of ERα in tumor cells compared to healthy tissue [47]. High expression of ERα and aromatase in GBM tissue samples was associated with the significantly longer survival of GBM patients, regardless of sex and body mass index [45]. The results also did not note any sex differences in ERα expression, confirming previous literature data [55].

In this study, we did not perform a comparison between patient-derived GBM tumors and healthy tissue as the latter was not available. The statistical analyses performed did not show differences in ERα expression between different tumoral areas at both the gene and protein levels. Therefore, the question arises as to why the result of the effect of hypoxia on the increase in ERα expression (obtained in an in vitro model) is not so clearly manifested in the results obtained from patient material. The answer may lie in the compensation mechanism and the continuous reciprocal equation of HIF-1 and ERα protein expression levels. It may also be a result of an overly general approach to the pool of tissues obtained from patients. Perhaps it would be correct to separate GBM tumors into different groups based on the level of expressed ERα expression in a manner similar to a study by Hönikl et al. [45].

It has been shown that one of the ERα isoforms, ERα-36, is particularly expressed in GBM tumor cells and shows expression in 96% of stage III–IV glioma samples, while it is very weakly expressed in stage I glial tumors [49]. The ERα-36 isoform is most often found throughout the cell, although expression can also be localized only in the nucleus or the cell membrane or the cytoplasm [49]. Similar observations for protein distribution throughout the cell volume were made for ERα expression in our study.

### 3.3. Estrogen Receptor β in a Model and In Vitro

We demonstrated lower *ERβ* mRNA expression under hypoxic and nutrient deficiency conditions in the U87 cell line. There was also no difference in ERβ protein expression and the degree of luminescence and cellular localization of ERβ protein between the test conditions. 

ERβ has been extensively studied in a number of cancers, including glial cell lines (T98G, U87, LN229, U138, M059J, M059K) in which mRNA and protein expression was found [43].

The present study did not examine the effect of ERβ expression on GBM cells; however, according to the literature, ERβ agonists induce a decrease in proliferation of glioma cell lines (T98G, U87, LN229, U138, M059J, M059K) [43,56] and inhibit the cell cycle in the G2/M phase [43]. ERβ agonists have also been shown to inhibit glial tumor growth in a xenograft model [56,57]. In addition, the use of ERβ agonists and selective estrogen receptor modulators inhibits glial tumor growth and promotes cell death by apoptosis [42,57]. Those results were achieved only in glial cell lines expressing ERβ, and the expression of the receptor itself increased when the ERβ agonist was injected into cell cultures [57].

In contrast to the aforementioned studies, tibolone, a selective tissue regulator of estrogen activity, has been shown to induce proliferation but not migration and invasion of GBM cell lines through the ER and to increase the expression of ERβ [44]. An increase in ERβ expression and silencing of ERα contributes to an increase in aquaporin two expression, thereby reducing the migratory capacity of GBM cells [58]. The effect of ERβ agonists through ERβ on processes such as proliferation or migration has been confirmed by determining the increase in expression of ERβ target genes [43]. Application of ERβ-specific siRNAs or shRNAs induces silencing of ERβ protein expression and abolishes the ability of ERβ agonists to reduce glioma cell proliferation [43].

Moreover, the combination of an ERβ agonist with TMZ has been found to be a good method of inhibiting glial tumor growth, probably through the effect of the ERβ agonist on inhibiting the activity of the PI3K/AKT/MTOR pathway, which contributes to the protection of tumor cells from TMZ-induced cytotoxicity [59]. 

ERβ, through a non-genomic mechanism involving the RAF/MAP2K1/ERK/ELK-1 signaling cascade (RAF, Raf kinases, ERK, extracellular signal-regulated kinases, ELK, ETS transcription factor, ETS transcription factor), increases the expression of the suppressor transcription factor EGR-1 (early growth response protein, EGR-1), which is involved in the regulation of cell growth, differentiation, and death. ETS transcription factor increased expression of the suppressor transcription factor EGR-1 (early growth response protein 1 (EGR-1)), which is involved in the regulation of cell growth, differentiation, and apoptosis [60].

In view of the above reports, we can assume a suppressive role for ERβ in the development of GBM. However, a decrease in *ERβ* mRNA expression under hypoxic and nutrient-deficient conditions reported in our study contradicts the literature data, where hypoxia, as well as HIF1α or HIF2α factors themselves, induce an increase in *ERβ1*, *ERβ2*, and *ERβ5* mRNA expression in U87 cells [61]. This discrepancy may be partly due to differences in the protocol used to induce hypoxia. The cited study used culture under oxygen-reduced conditions, whereas our study used PDH inhibition. On the other hand, the results for ERβ protein expression were consistent with the literature, e.g., with the study by Habib et al. in which the authors also observed no differences in ERβ protein expression in cells exposed to hypoxia [52].

The aforementioned results on ERβ appear to be contradictory because subjecting cells to stress conditions such as hypoxia and nutrient deprivation should theoretically induce an increase in the expression of anti-proliferative factors. However, the work of Attwood et al. shows that the introduction of the selective ER modulator raloxifene into cell cultures delays the dissolution of stress granules. Stress granules, formed as a result of stress conditions for GBM cells, are clusters of mRNAs and proteins required for normoxia. Translation of these mRNAs begins after the stress conditions cease and the removal of these granules is necessary for the cell to readapt to normoxia [62]. Assuming that ER activation contributes to prolonging the time for a cell to revert to its invasive phenotype, this may explain a decrease in ERβ expression under stress conditions.

### 3.4. Estrogen Receptor β in Tumors Taken from Patients 

In this study, *ERβ* mRNA expression was lower in the tumor core than in the tumor cortex overall and after sex division in men. We also found that in patient samples, ERβ protein expression was lower in the core than in the periphery of the tumor overall and after sex division in women. With immunohistochemistry, we found that ERβ protein expression was highest in the tumor cortex, followed by the tumor periphery, and lowest in the tumor core.

The occurrence of ERβ has been described in astrocytic gliomas [63] and in oligodendrogliomas [64], where ERβ-positive patients showed longer survival [64]. ERβ is also present in healthy astrocytes, and as tumor malignancy increases, the expression of this receptor decreases in the tumor [43,55,63,64,65]. Expression in healthy astrocytes was also shown to be lower than in glial tumors [55], which somewhat contradicts our results. Although we did not compare tumors with healthy tissue, the expression of ERβ protein was lower in the peritumoral area which was partly composed of healthy tissue. It should be noted, however, that these results may be due to the presence of a part of the tumor with high expression of ERβ protein in the peritumoral area, which may interfere with the reading for normal tissue. This hypothesis is supported by a recently published study describing higher levels of ERβ expression in GBM compared to healthy tissue and low-grade gliomas [47]. The higher expression of ERβ can also result from the determination of the expression of all isoforms, the major isoform of ERβ in glioma being ERβ5, whose expression is higher in tumors than in healthy tissue [61] and tends to increase with the degree of tumor malignancy [61]. The suppressor function of ERβ demonstrated in other types of cancer is also found in glioma, which is supported by a report suggesting that ERβ is not involved in the epithelial–mesenchymal transition process that is characteristic of GBM [47].

ERβ protein expression in this study was higher in the peritumoral area than in the tumor core, which is consistent with the suppressive function of ERβ and the fact that cells in the peritumoral area have a lower proliferative potential than those in the enhancing tumor region. However, as our results describe the expression of ERβ without subdividing it into isoforms, this may make interpretation difficult, especially given the incomplete concordance of published reports.

ERβ5 has been found to inhibit oncogenic pathways such as PI3K/AKT/mTOR and MAPK/ERK in a ligand-independent manner [61]. However, the findings by Liu et al. contradict these reports, depicting the ERβ1 isoform as a tumor suppressor in GBM, and the ERβ5 isoform as pro-oncogenic in GBM [66]. It has also been shown that the use of an epigenetic modulator of *ERβ* expression, a histone deacetylase inhibitor, induces an increase in the expression of the ERβ1 isoform, which acts as a tumor suppressor, but not the ERβ5 isoform, which drives the oncogenic function [67]. However, the use of a histone deacetylase inhibitor in combination with an ERβ agonist induces a strong reduction in cell viability, invasion, colony formation, and increased apoptosis [67]. The aforementioned studies confirm that GBM is characterized by low ERβ expression, probably caused by silencing of the *ERβ* gene through hypermethylation [54].

In contrast, the *ERβ* expression presented in our study indicates an increase in mRNA expression in the enhancing tumor region compared to the tumor core. The results presented here confirm data from an in vitro model where stress conditions such as hypoxia and nutrient deficiency in the tumor core may contribute to a decrease in *ERβ* mRNA expression. 

In a study by Sareddy et al., the localization of the ERβ in the cell varied depending on the malignancy grade of the glial tumor. In grade II tumors, ERβ was mainly localized in the nucleus, whereas in tumors with a high degree of malignancy, ERβ was mainly localized in the cytoplasm. The percentage of cells with nuclear staining decreased with the degree of malignancy [43]. Although in our study, ERβ expression was evident in both the nucleus and cytoplasm, significantly more cells showed the cytoplasmic staining positivity characteristic of highly malignant glial tumors, which includes GBM.

In addition, a study by Kefalopoulou et al. showed that the expression of ERβ coactivators, such as AIB1 (nuclear receptor coactivator 3 amplified in breast 1), TIF2 (nuclear receptor coactivator 2), and PELP1 (proline-, glutamic acid-, and leucine-rich protein 1), are involved in the initiation, progression, and metastatic potential in various types of cancer [68]. The described expression of ERβ coactivators, however, was inversely correlated with the expression of ERβ [68].

In the analyses presented here, no differences in ERβ expression were found between males and females, confirming the available literature data, where no sex-related differences in ERβ protein expression were found in glial tumors (24 staphylomas and 8 gliomas) [55]. In the overall analysis of glial tumors, the authors found a borderline statistically significant higher expression of ERβ protein in women (no result was reported for GBM alone) [65]. However, the *p*-value was 0.058, which is a tangentially insignificant value by generally accepted standards, including in our paper. Of course, one cannot deny a certain trend here, which may to some extent confirm the difference in ERβ expression after accounting for sex in the present study. The observed discrepancy in ERβ mRNA expression between the enhancing tumor region and the core of the tumor, after taking sex into account, was found only in men. In contrast, the difference in ERβ protein expression between the core and the peritumoral area was observed only in women. Although the differences are difficult to interpret given the current knowledge of ERβ expression in GBM, they may represent an expression of as yet undescribed relationships, leading us to conclude that further research is needed to determine the detailed role of ERβ in GBM.

## 4. Materials and Methods

In the present study, we used a research scheme from a previously published paper: Androgen Receptor Expression in the Various Regions of Resected Glioblastoma Multiforme Tumors and in an In Vitro Model, by Simińska et al. [69].

### 4.1. In Vitro Model: Cell Culture

Two research models were used in the study. The in vitro model was established using a cell culture of the U87 line to test whether factors such as hypoxia, nutrient deficiency, and necrosis that affect the formation of different areas in the tumor alter the expression of estrogen receptors. A detailed description of the culture conditions is presented in Simińska et al. [69]; this article provides a summary of the procedure.

Cell culture of the U87 line (obtained from the European Collection of Authenticated Cell Cultures (ECACC)) was performed under standard conditions of 37 °C, 95% humidity, and 5% CO_2_, according to the manufacturer’s instructions. Cells were seeded at a density of 20,000/cm^2^ in 6-well culture plates (Nest, Scientific Biotechnology, Wuxi, China) and cultured for 3 days until appropriate confluence. Then, cells were cultured in control conditions according to the manufacturer’s instructions (EMEM (Sigma-Aldrich, Poznań, Poland), 10% FBS (inactivated fetal bovine serum) (Gibco Limited, Brigg, UK), 100 U/mL penicillin (Gibco Limited, Brigg, UK), 100 µg/mL streptomycin (Gibco Limited, Brigg, UK), and 1% non-essential amino acid (Sigma-Aldrich, Poznań, Poland)). In a cell cultured in hypoxic conditions, 100 µM cobalt chloride (Sigma-Aldrich, Poznań, Poland) was added to the standard medium. Cells grown in nutrient-deficient conditions had their glutamine concentration reduced to 0.2 mM L-glutamine (Sigma-Aldrich, Poznań, Poland) and were deprived of sodium pyruvate. In a cell grown in necrotic conditions, 200 µM hydrogen peroxide (Sigma-Aldrich, Poznań, Poland) was added to the medium. Culture in control and experimental conditions was carried out for 24 h and was performed in 6 repetitions for each of the tested conditions. The cultures obtained in this way were collected for gene and protein expression studies of the tested estrogen receptors. In a similar way, a culture was prepared for confocal analysis with the additional step of placing slides coated with sterile-filtered 0.01% poly-l-lysine solution (BioReagen, Sigma-Aldrich, Poznań, Poland) in a plate.

### 4.2. Model of Tumoral Areas of Patients’ GBM Tumors

In the second research model (a detailed description of the model is given in Simińska et al. [69]), material taken from different areas of the patient’s tumor (tumor core, enhancing tumor region, and peritumoral area) was introduced to show differences in estrogen receptor expression in different areas of the tumor.

The patients included in the project were patients of the Department of Neurosurgery and Pediatric Neurosurgery of the Pomeranian Medical University in Szczecin, Poland. The research project received consent from the Bioethics Committee of the Pomeranian Medical University in Szczecin, resolution No. KB0012/96/14/A-1 of 9 March 2020. All recruited patients were familiarized with the conditions and assumptions of the study and informed consent was obtained from all of them. Participation in the project did not change the treatment regimen that the patient underwent. The number of patients qualified for the presented study was 24, including 14 men and 10 women. The average age of the study group was 62 years; all qualified people were adults. All tumors of qualified persons had a histopathological diagnosis of glioblastoma multiforme without mutations in the *IDH* (isocitrate dehydrogenase) gene. During the surgery to remove the GBM tumor, three tumor areas were collected from the tumor (tumor core, enhancing tumor region, and peritumoral area). The tumor core is the non-growing central area of the tumor, characterized by hypoxia and pseudopalisade necrosis, resulting from insufficient blood supply to these regions of the tumor and, therefore, low access to oxygen and nutrients. The enhancing tumor region is an area of intense tumor growth; in this area there is high proliferation and an increase in tumor mass. The peritumoral area is an area of healthy tissue partially infiltrated by tumor cells. Due to the presence of cells migrating along Scherer’s structures in this area [70], it is also often called a niche invasion. The identification of the above areas in the patients’ tumors was possible thanks to the neuronavigation method used during surgery.

### 4.3. Quantitative Real-Time Polymerase Chain Reaction (qRT-PCR)

For both research models, gene expression analysis of *ESR1* and *ESR2* was performed in a manner similar to the previous work [69]. In this paper, we briefly describe the research techniques used.

To perform qRT-PCR analysis, mRNA isolation was performed from the obtained material. The RNeasy Lipid Tissue Mini Kit (Qiagen, Hilden, Germany) was used for the tumor areas of the GBM tumor and the RNeasy Mini Kit (Qiagen, Hilden, Germany) for the cell culture material. The concentration and purity of the obtained isolate were checked using a Nanodrop ND-1000 (Thermo Fisher Scientific, Waltham, MA, USA). In a further step, the mRNA was transcribed into cDNA using Reverse Transcription PCR. qRT-PCR analysis was performed using a Power SYBR Green PCR Master Mix (Applied Biosystems, Thermo Fisher Scientific, Waltham, MA, USA) and an ABI 7500 analyzer (Applied Biosystems, Thermo Fisher Scientific, Waltham, MA, USA) (95° C (15 s), 40 cycles of 95 °C (15 s) and 60 °C (60 s)). The following primer sequences were used: ESR1: CCCACTCAACAGCGTGTCTC |CGTCGATTATCTGAATTTGGCCT, ESR2: AGATTCCCGGCTTTGTGGAG |GAGCAAAGATGAGCTTGCCG. The expression level of the glyceraldehyde-3-phosphate dehydrogenase (*GAPDH*) gene was selected as an endogenous control. The method was performed in the same way as described in the work by Simińska et al., where it is described in more detail [69].

### 4.4. Enzyme-Linked Immunosorbent Assay (ELISA)

The levels of ERα and ERβ proteins were also determined for both models using ready-to-use ELISA-type kit reagents. The same tissue preparation methodology was used as previously described [69], the kits used were from the same manufacturer: Human ERα ELISA Kit (EH0033) (FineTest, Wuhan, China), Human ERβ ELISA Kit (EH3015) (FineTest, Wuhan, China).

Material from GBM tumors and cell cultures was homogenized according to the manufacturer’s recommendations of the ELISA test kits used, consisting of knife homogenization in PBS (0.01 M, pH = 7.4) (SigmaAldrich, Poznań, Poland) with proteinase inhibitors (PhosSTOP and cOmplete, Mini Protease Inhibitor Cocktail, Sigma-Aldrich, Poznań, Poland). The homogenized material was then centrifuged (5 min at 5000× *g*) and the supernatant was collected, in which the total protein concentration was determined using MicroBCA-Pierce™ (Thermo Fisher Scientific, Waltham, MA, USA). The supernatant was diluted to the dilution recommended by the manufacturer (0.3 mg/mL) and the ELISA tests given above were performed. The results were read using a plate reader (BiochromAsys UVM 340, Biochrom, Cambridge, UK), and the obtained concentrations were normalized to the amount of total protein in the sample.

### 4.5. Immunohistochemistry

Immunohistochemistry was performed on patients’ tumor tissues, analogously to the previous work [69].

Tumor fragments were properly fixed in formaldehyde, embedded in paraffin blocks and cut using a microtome (Microm HM340E Thermo Fisher Scientific, Waltham, MA, USA), into sections placed on polylysine-coated slides (Sigma-Aldrich, Poznań, Poland). The preparations were then deparaffinized and rehydrated. In the next step, they were boiled twice in 10 mM citrate buffer at pH 9.0 (Dako Inc. Canpinteria, CA, USA) (4 and 3 min in a microwave oven (700 W)). Peroxidase blocking was further carried out using the Dako LSAB + System kit -HRP (Dako Inc., Canpinteria, CA, USA) (10 min at room temperature). Further overnight incubation was performed with ERα Antibody (sc-8005, Santa Cruz Biotechnology, Dallas, TX, USA) and ERβ Antibody (sc- 390243, Santa Cruz Biotechnology, Dallas, TX, USA) (separately, dilution 1:50). Then, the slides were stained using reagents from the manufacturer’s kit Dako LSAB + System-HRP (Dako Inc., Canpinteria, CA, USA) and hematoxylin. Photos of the preparations were taken using a Leica DM5000 B light microscope (Leica, Wetzlar, Germany) integrated with a camera. In the analyzed preparations, photos were taken in triplicate in representative places (necrotic area, enhancing tumor area, peritumor area). All cells visible in the images were summed and the percentages of ERα- and ERβ-positive cells were counted.

### 4.6. Confocal Microscopy

For the in vitro model, analysis was performed using confocal microscopy analogously to the previous work [69].

In the first stage, cell culture preparations were permobilized in 0.5% TRITON ×100 solution (Sigma-Aldrich, Poznań, Poland) (20 min). The slides were further blocked for another 20 min using blocking serum (2.5% horse serum in PBS (Thermo Fisher Scientific, Waltham, MA, USA). The slides were further incubated for 1 h at room temperature in a humid chamber with ERα Antibody (sc-8005, Santa Cruz Biotechnology, Dallas, TX, USA) and ERβ Antibody (sc-390243, Santa Cruz Biotechnology, Dallas, TX, USA) (separately). The preparations were then incubated with fluorochrome-conjugated II-row antibody (FITC Merck Millipore, Poznań, Poland) for 1 h at room temperature in a humid chamber in the dark. In the next stage, incubated with DAPI (Merck Millipore, Poznań, Poland) (20 min, room temperature). Then, they were sealed in a fluorescence mounting medium (Dako Inc., Canpinteria, CA, USA). The preparations thus obtained were assessed and photographed using an FV1000 confocal microscope (Olympus, Hamburg, Germany) in combination with an IX81 inverted microscope (Olympus, Hamburg, Germany). Images were recorded using a 488 nm laser for FITC and a 405 nm laser diode for DAPI.

### 4.7. Statistical Analysis

Statistical analysis was performed using Statistica software (version 13, StatSoft Poland, Krakow, Poland) in a manner analogous to that previously described in Simińska et al. [69]. Generally, the Shapiro–Wilk test was performed to check the normal distribution of the obtained data; in the case of the cellular model, normal distributions were obtained, while in the model from different areas of GBM tumors obtained from patients, normal and abnormal distributions were obtained. For the data obtained from the cell model, a T-test was then performed to determine the differences between the induced test conditions and the control. The Mann–Whitney U-test and Wilcoxon signed-rank test were performed for data obtained from tumor areas obtained from GBM tumors. The first one was used to compare identical areas between a group of women and a group of men. The second one was used to compare the tumoral areas with each other. Values of *p* < 0.05 were considered statistically significant.

## 5. Conclusions

Our results suggest that conditions (especially hypoxia) that persist in individual tumor areas influence the expression of the estrogen receptors studied. In the future, more detailed analyses should be performed to assess the expression of individual estrogen receptor isoforms in the three areas of the GBM tumor.

### Research Limitations

The patient group was homogeneous in terms of ethnicity, so the results obtained in the study may be specific to patients from this region. However, relevant comparative analyses were not performed due to lack of access to another patient pool. 

Due to technical limitations in obtaining a larger number of patients (one clinical center in the region, the health condition of patients before surgery making it difficult to obtain informed consent to participate in research and collecting material in each case, the number of patients being limited due to the low incidence of GBM, and the lack of target histopathological diagnosis in each case considered), the group of patients included in the study was relatively small.

The hypoxia model used does not fully reflect the hypoxic conditions prevailing in the tumor. However, cobalt chloride produces the biochemical effects observed in hypoxia in cultured cells.

Our studies were performed only on one U87 cell line as a GMB tumor model. However, this line is widely used as a research model (although it also has its limitations) of this cancer and it allowed us to compare our research results with those of other research teams, but the use of other lines would certainly enrich our research.
